# Supplementary material for: Comparative Genomic Analysis of Antimicrobial-Resistant Escherichia coli from South American Camelids in Central Germany
Source: Microorganisms. 2022 Aug 24;10(9):1697. doi: 10.3390/microorganisms10091697 (PMC9501560; doi:10.3390/microorganisms10091697)
Supplement: Supplementary file 1 [file microorganisms-10-01697-s001.zip › TS2_Supplemental_data_AMR.pdf]

Antibiotic class	ARG Total	%	ARG	No.	%
**Beta-lactam**	35	90%	blaCMY-2	2	5%
			blaCTX-M-15	2	5%
			blaCTX-M-1	16	41%
			blaCTX-M-27	2	5%
			blaCTX-M-65	1	3%
			blaOXA-10	1	3%
			blaSHV-12	1	3%
			blaTEM-1B	18	46%
**Fluoroquinolone**	24	62%	qnrB19	1	3%
			qnrS1	4	10%
**Fluoroquinolone mutations**			gyrA:p.S83L	22	56%
			parC:p.S80I	19	49%
			gyrA:p.D87N	17	44%
			parC:p.A56T	3	8%
			parC:p.A56T	1	3%
**Folate pathway antagonist**	22	56%	dfrA8	1	3%
			dfrA12	1	3%
			dfrA14	4	10%
			dfrA17	9	23%
			dfrA1	4	10%
			sul1	6	15%
			sul2-2	12	31%
			sul2-3	5	13%
			sul3	2	5%
**Aminoglycoside**	20	51%	aac(3)-Iid	2	5%
			aadA1	1	3%
			aadA2	4	10%
			aadA5	10	26%
			ant(3'')-Ia	5	13%
			aph(3'')-Ib-1	4	10%
			aph(3'')-Ib-5	13	33%
			aph(3'')-Ia-1	2	5%
			aph(6)-Id	15	38%
**Tetracycline**	18	46%	tetA(6)	10	26%
			tetB(1)	2	5%
			tetB(2)	8	21%
**Phenicol**	12	31%	catA1	5	13%
			cmlA1	2	5%
			floR2	6	15%
**Macrolide**	7	18%	mefC	2	5%
			mphG	2	5%
**Macrolide/Rifamycin**			mphA	4	10%
**Rifamycin**	1	3%	arr-3	1	3%
